# Lifetime and Child Sexual Violence, Risk Factors and Mental Health Correlates Among a Nationally Representative Sample of Adolescents and Young Adults in Haiti: A Public Health Emergency

**DOI:** 10.1177/08862605221102484

**Published:** 2022-05-16

**Authors:** Jude Mary Cénat, Rose Darly Dalexis, Lewis Ampidu Clorméus, Marie-France Lafontaine, Mireille Guerrier, Guesly Michel, Martine Hébert

**Affiliations:** 1 School of Psychology, 6363 University of Ottawa, Ontario, Canada; 2 Interdisciplinary School of Health Sciences, 6363 University of Ottawa, Ontario, Canada; 3 Faculty of Ethnology, 354984 State University of Haiti, Port-au-Prince, Haiti; 4 Université Libre d’Haïti, Cap-Haitian, Haiti; 5 Department of Sexology, Université du Québec à Montréal, Québec, Canada

**Keywords:** lifetime sexual victimization, child sexual victimization, traumas, mental health problems, depression, posttraumatic stress disorder, psychological distress, substance abuse

## Abstract

Very little is known in Haiti and the Caribbean regarding child and lifetime sexual victimization. Using a nationally representative sample of adolescents and young adults aged 15–24, this study aimed to document the prevalence, risk factors and mental health correlates of lifetime and child sexual violence in Haiti. A national cross-sectional surrvey was conducted in Haiti, using a multistage sampling frame, stratified by geographical department, urban or rural setting, gender, and age groups (15–19 and 20–24 years). The final sample included 3586 household participants (47.6% female). A weighted sample of 3945 individuals was obtained and used in the following analyses. Overall rate of lifetime and child sexual violence was, respectively, 27.44% (95% CI 25.94–28.94) and 11.27% (95% CI 10.18–12.35). Lifetime sexual violence rate was significantly higher among female participants (29.02%; 95% CI 27.5–30.55) compared to male (25.73%, 95% CI 24.26–27.2), χ^*2*^ = 4.63, *p* < .05, but there was no significant gender difference for child sexual victimization. Experiences of family physical violence, emotional abuse by mother and father, divorce of parents, and other physical violence were strongly associated with higher odds of sexual victimization. Participants who reported having experienced sexual violence are more at risk to meet criteria of PTSD (OR = 1.96, 95% CI 1.66–2.32; *p* < .0001), depression (OR = 1.73, 95% CI 1.47–2.02; *p* < .0001), psychological distress (OR =1.72, 95% CI 1.47–2.02; *p* < .0001), and substance abuse (OR = 1.33, 95% CI 1.13–1.57; *p* < .0001). Findings demonstrate that sexual violence is a public health emergency in Haiti. They provide evidence for the development of prevention and intervention programs.

## Introduction

Sexual violence is a major global public health concern and constitutes a barrier for the development of low- and middle-income countries (Bowman et al., 2008; Dubowitz, 2017; Finkelhor, 1994; Krug et al., 2002; Stoltenborgh et al., 2011; WHO, 2013). While sexual violence remains a global human rights and public health problem, adolescents, and young adults in low- and middle-income countries (LMICs) are particularly vulnerable (Decker et al., 2015; Veenema et al., 2015). Studies have shown that close to one-third of youth in LMICs have experienced at least one episode of sexual violence in their childhood (Reza et al., 2009; Veenema et al., 2015). Studies conducted in some African countries such as South Africa, Tanzania, Kenya, and Swaziland showed a prevalence rate of sexual violence varying between 9.9% and 37.8% (Averdijk et al., 2011; Gladden et al., 2011; Reza et al., 2009; United Nations Children’s, 2012; Ward et al., 2018). They also found that girls and young women are more at risk to experience sexual violence than boys and young women (Averdijk et al., 2011; Gladden et al., 2011; Reza et al., 2009; United Nations Children’s, 2012; Ward et al., 2018). Although the sexual violence phenomenon is less studied in the Latin American region, the few studies showed that it constitutes a major health and social issue among children, adolescents, and young adults (Contreras Taibo et al., 2020; López et al., 2017; Wang et al., 2017). In the Caribbean, few studies have explored sexual violence in representative samples of youth and even fewer assess the prevalence of sexual violence and explore the associated consequences in adolescents and young men (Debowska et al., 2018; Descartes et al., 2020; Jones, 2021; Lai et al., 2020; Reid et al., 2014).

## The Haitian Context

Haiti, the least developed country in the Caribbean and in the Western Hemisphere, is also affected by the major global health problem which is sexual violence (Gilbert et al., 2018; Lai et al., 2020; Sumner et al., 2015). With more than 50% of the national population under the age of 24, studies showed that adolescent girls and young women are particularly at risk of being victims of sexual violence (Cénat et al., 2020a; Kolbe et al., 2010; Lai et al., 2020; Sumner et al., 2015). A study conducted in the post-earthquake context as part of the Violence Against Children Surveys and including displaced people showed that 23.01% of adolescent girls and young women and 15.98% of adolescent boys and young men aged 13–23 had experienced sexual violence (Lai et al., 2020). Another publication using the same data reported that 9% of young women and 7.6% of young men aged 18–24 had experienced coerced and forced sex before the age of 18 (Sumner et al., 2015). However, no study has provided details on the different forms of sexual violence and their associated consequences have been studied in combination with other forms of violence (physical and emotional) encountered by these youth (Lai et al., 2020). In addition, to this point, no study has explored risk factors, and consequences associated with sexual violence among a representative sample of male and female adolescents and young adults in Haiti. Studies around the world have also shown that sexual violence has significant consequences on the physical and mental health of victims, in the short, medium, and long term (Hébert et al., 2020; Jonas et al., 2011; Sanjeevi et al., 2018; Walker-Descartes et al., 2021; Ward et al., 2018).

## The Present Study

Face with the critical lack of data in Haiti on sexual violence, information on the prevalence, risk factors, and consequences of sexual violence among adolescent girls and young women as well as adolescent boys and young men is clearly needed to help inform the development of prevention and intervention programs to eradicate this major public health problem. The present study aims to provide these findings by assessing: 1) the prevalence of lifetime sexual violence among adolescents and young women and men aged 15–24 years depending on the identity of the perpetrator and sociodemographic characteristics; 2) the prevalence of child sexual victimization; and 3) the risk factors (divorce of parents, physical victimization, family physical violence, emotional abuse by mother, emotional abuse by father, gender and other sociodemographic characteristics) and mental health consequences (PTSD, depression, psychological distress and substance abuse) associated with sexual victimization. Gender is compared at each level of the analyses.

## Methods

### Procedure and Sampling

We used a multistage stratified sampling approach to collect and analyze data within the Haitian population aged 15–24 years on sexual violence involving contact and committed by different perpetrators and various health conditions. Participants were sampled in the 10 geographic departments of Haiti. In each geographic department, households were selected based on two areas of residence: rural or urban. In each area (rural or urban), the first household was selected randomly by the team coordinator, starting from the village or city entrance or the central place. Then, depending on the density of the houses, the other households were selected (between every three houses to every 12 houses). Participants also included street youth who were randomly selected in two service centers in Port-au-Prince. Some youth found in households also defined themselves as living on the street. This is understandable given the different categories of street children in Haiti (Cénat et al., 2018; Derivois et al., 2017). The sample does not include adolescents or young adults in prison, which remains very marginal in Haiti. Within each household, one participant was randomly selected based on gender (female and male), and the two target age groups (15–19 and 20–24 years). Interviews were performed from September to October 2020. Participants were eligible to participate if they were a resident of a selected household in a given residence area/geographical division and were between 15 and 24 years old. Prior to completing the questionnaire, participants gave their written informed consent. The research protocol was approved by the Research Ethics Boards of the University of Ottawa and the Université Libre d'Haïti.

The questionnaire was translated in Haitian creole (translation-back translation) according to World Health Organization guidelines for measure’s translation (World Health Organization, 2017) and adapted to the cultural, social and educational context of Haiti by a panel of 7 experts from different fields (psychology, social work, applied linguistics, women’s studies, and sociology). It was also available in French. Interviewers were trained during 3 days and benefited on-site support throughout the survey from supervisors. All interviewers are fourth-year students in social sciences at the State University of Haiti. The training addressed gender, cultural, social, educational aspects and biases, ethical issues, way to establish rapport to encourage disclosure. Interviewers went door-to-door in selected households to invite potential participants to participate in the survey. When no one was present in the selected household or no one wanted to participate, the next household was selected. The participation rate was 76.31%. Interviews were conducted in private space in respect of confidentiality.

A total of 3635 participants responded to the survey; 49 did not meet the eligibility criteria (completely missing data and out of the desired age range) and were excluded from the sample. The sample consisted of 3586 participants (47.6% female) between the ages of 15–24 (*M* = 19.37 years, *SD* = 2.71). Close to half (51.6%) of the participants were between 15 and 19 years old and 48.4% 20–24 years old. Table 1 presents the sociodemographic characteristics of the sample.

**Table 1.** Sociodemographic Characteristics of the sample (*n* = 3944).

	Age Group			
	15–19 (*n* = 1,966)		20 and more (*n* = 1,978)	
Variable	*n*	%	*N*	%
Gender				
Female	1015	51.6	1027	51.9
Male	951	48.4	951	48.1
Other	0	0	0	0
Residence area				
Rural	1133	57.6	1001	50.6
City	834	42.4	977	49.4
Marital status				
Single	1745	92.1	1721	89.0
Married	22	1.2	47	2.4
In a relationship	64	3.4	56	2.9
Divorced	0	.0	5	.3
Widowed	6	.3	8	.4
Separated	13	.7	16	.8
Other	44	2.3	80	4.1
Employment				
No	1792	93.0	1654	85.0
Yes	135	7.0	293	15.0
Children				
No	1869	96.5	1766	90.6
Yes	68	3.5	184	9.4
Religion				
None	202	10.5	135	6.9
Catholic	501	25.9	478	24.3
Voodooist	79	4.1	125	6.4
Baptist	551	28.5	575	29.2
Pentecostal	196	10.2	299	15.2
Methodist	49	2.5	42	2.1
Episcopal	10	.5	33	1.7
Muslim	2	.1	4	.2
Mormon	11	.6	10	.5
Adventist	150	7.8	136	6.9
Jehovah’s witness	30	1.6	34	1.7
Other	150	7.8	97	4.9
Parent’s marital status				
Married	855	45.3	987	51.1
In a relationship	223	11.8	260	13.5
Divorced	56	3.0	52	2.7
Widowed (one or both deceased)	227	12.0	231	12.0
Separated	505	26.7	380	19.7
Other	23	1.2	21	1.1
Father’s employment status				
Employed	1026	54.3	930	49.2
Unemployed	862	45.7	960	50.8
Mother’s employment status				
Employed	729	38.8	728	38.6
Unemployed	1149	61.2	1157	61.4

Sampling weights were computed and applied to data to be able to produce estimates representative of the population of interest and compensate for any selection biases. Sampling weights were obtained utilizing the selection probabilities in the current survey and population. Data from the Mortality, Morbidity, and Service Utilization Survey conducted in conjunction with The Demographic and Health Survey (DHS) program in 2016–2017 were used to calculate population-based selection probabilities (RABQ et al., 2012). This survey is the most recent study of its kind conducted in Haiti due to the unavailability of recent census data in that country. Given its solid methodology, it constitutes a representative sample of the population.

Using cross tabulation, the probabilities of an individual being selected from the population (DHS data) were obtained by considering these characteristics: 10 geographical divisions, areas of habitation (urban or rural), age groups (15–19 and 20–25 years), and gender (boys/men and girls/women); the same was calculated in the current sample. Sampling weights were calculated as a ratio of the two, that is, the probability of choosing an individual from the population with the given set of characteristics divided by the probability of choosing the same individual from the current sample (Johnson, 2008; Kalton & Flores-Cervantes, 2003). A weighted sample of 3945 individuals was obtained and used in the following analyses. Other details are provided in a previous publication (Cénat et al., 2022). Table 1 presents the sociodemographic characteristics of the sample.

## Measures

### Sociodemographic Questionnaire

Sociodemographic information was collected: sex, age, education level, area of residence, living place, marital status, parents’ marital status, and employment status.

### Sexual Victimization

Two items on the sexual violence scale were drawn and adapted from measures used in previous surveys among children and adolescents (Finkelhor et al., 1990; Tourigny et al., 2008). The scale assessed the presence of unwanted sexual contacts that had occurred in their lifetime. The possible perpetrators in this scale were adapted to the Haitian population’s needs (e.g., sport coach was replaced by religious leader), The first question measured unwanted sexual contact without penetration (e.g., fondling, touching): “*Have you ever been touched sexually when you did not want to, or have you ever been manipulated, blackmailed, or physically forced to touch sexually…*.” The participants responded to the items on a dichotomous scale (1 = yes and 2 = no) for each of these perpetrators: (1) an immediate or extended family member, (2) a teacher, (3) a religious leader (e.g., pastor, priest, etc.), (4) a known person outside your family (other than a romantic partner), and (5) a stranger. Then, participants aged 18–24 years old were asked if this happened before 18 years old (item 6). The second item assessed unwanted sexual contacts with penetration (i.e., oral, anal, or vaginal) “*Excluding the sexual touching mentioned in the previous item, has anyone ever used manipulation, blackmail, or physical force, to force or obligate you to have sex (including all sexual activities involving oral, vaginal, or anal penetration) with…*” and was answered the same way as the first question for perpetrators (items 7, 8, 9, 10, and 11). Then, participants aged 18–24 years old were asked if this happened before 18 years old (item 12). The measure of sexual violence specifically precluded sexual violence involving a romantic partner from its measurement. The different forms of unwanted sexual contacts and responses on type of perpetrators were coded as 1 for the presence of sexual violence and 0 for the absence of victimization.

### Physical Violence

A single yes or no question from the Life Events Checklist for DSM-5 and widely used previous to the DSM-5 in similar studies have been used to assess physical violence (Bremner et al., 2007; Finkelhor et al., 1990; Weathers et al., 2013): “Have you ever been experienced physical assault (for example, being attacked, hit, slapped, kicked, beaten up)?”

### Violence from Parents

We used a 3-item measure to assess physical (To punish me, my parents always used force (whipping, beating, hitting, etc.) and emotional (…tells me hurtful and/or insulting things) violence from mother and father (Cénat et al., 2019). Items ranged on a 5-point scale from 0 (never) to 4 (very often). This measure was categorized as yes (rarely to very often) or no (Never). The Cronbach’s alpha in our sample was .91.

### Posttraumatic Stress Disorder

The PTSD Checklist for DSM-5 (Weathers et al., 2013) is a 20-item self-report questionnaire that assesses the 20 symptoms of PTSD present in the DSM-5 (e.g., “*In the past month, how much were you been bothered by repeated, disturbing, and unwanted memories of the stressful experience?*”). Responses are reported on a 5-point scale: (0) Not at all, (1) A little bit, (2) Moderately, (3) Quite a bit, and (4) Extremely. The PCL-5 has a strong internal consistency (α = .94), test-retest reliability (r = .82), and convergent (rs = .74 to .85) and discriminant (rs = .31 to .60) validity (Blevins et al., 2015). A cutoff score of 33 to meet criteria of PTSD was suggested (Ashbaugh et al., 2016; Blevins et al., 2015; Sveen et al., 2016). The Cronbach’s alpha in our sample was .97.

### Depression

The Centre for Epidemiological Studies Depression Scale (CES-D-10) measures depressive symptoms (Andresen et al., 1994). The scale includes 10 items on a 4-point scale (*Rarely or none of the time* to *All of the time*). The overall score ranges from 0 to 30; higher scores indicate more severe symptoms (e.g., “I felt that everything I did was an effort”). This scale has demonstrated excellent divergent (.89) and convergent (.91) validity, as well as excellent reliability (.85) (Björgvinsson et al., 2013; Miller et al., 2008; Radloff, 1977). The 10 items are summed to get a total score. Items 5 and 8 must be scored in reverse. The optimal cut score for the CES-D-10 that indicates meeting criteria for depression symptoms is 10 (Zhang et al., 2012). The Cronbach’s alpha in our sample was .81.

### Psychological Distress

We assessed psychological distress using the 10-item version of the Kessler Psychological Distress Scale (Kessler et al., 2002). The scores are calculated by summing the items endorsed on a 5-point Likert scale (0–40): (0) Never, (1) Rarely, (2) Sometimes, (3) Most of the time, (4) All the time. A score of 12 and above represents a clinical score of psychological distress (Boak et al., 2014). The Cronbach’s alpha in our sample was .89.

### Substance Abuse

Substance violence was assessed using three items from the *Screening Grid for Detection of Alcohol and Drug Problems in Adolescents* (Landry et al., 2004). The measure assesses the consumption frequency of alcohol, cannabis, and other drugs (e.g., ecstasy, amphetamine, speed, cocaine, and acid): “in the past 12 months, how many times have you consumed these products?”. The items are scored on a 5-point scale: (0) *Not at all*; (1) *Occasionally*; (2) *About once a month*; (3) *On weekends or once or twice a week*; (4) *3 times a week or more*; and (5) *Every day*. A dichotomized score was calculated based on whether participants used these substance one to two times a week versus three times a week or more (Cénat, Blais, et al., 2018). The Cronbach’s alpha in our sample was .87.

## Data Analysis

We documented the prevalence of different forms of sexual violence along with gender difference using chi square test. Logistic regressions were performed to assess the associations between sexual violence and correlates including other forms of victimization and violence exposure, protective and risk factors, and mental health consequences. Unadjusted and adjusted odds ratio (OR) were estimated where unadjusted OR was derived from a model including a specific correlate at a time while adjusted odds ratio resulted from a model integrating the concurrent effect of the whole group of correlates. Sampling weights as described above were applied to the data. Analyses were performed using Statistical Package for Social Science (SPSS—version 26, IBM, USA).

## Results

Overall rate of lifetime sexual violence was 27.44% (95% CI 25.94–28.94) (Table 2). Lifetime sexual violence prevalence was significantly higher among female participants (29.02%; 95% CI 27.5–30.55) compared to male (25.73%, 95% CI 24.26–27.2), χ^*2*^ = 4.63, *p* < .05. Also, 25.66% (95% CI 24.19–27.13) of participants reported that they experienced sexual violence without penetration, with a higher prevalence for female participants (27.21%, 95% CI 25.71–28.7) compared to male participants (24%, 95% CI 22.57–25.44), χ^*2*^ = 4.55, *p* < .05. The prevalence of sexual violence involving penetration was 14.47% (95% CI 13.25–15.69) with the same pattern, that is, significant difference between female participants (15.65%, 95% CI 14.39–16.91) compared to male (13.19%, 95% CI 12.01–14.36), χ^*2*^ = 3.93, *p <* .05.

**Table 2.** Prevalence of lifetime sexual victimization over type of perpetrators, and child sexual victimization (*n total* = 3944; *n 18 and +* = 2866).

	Female Participants			Male Participants			Total			Chi Square* (*p*-value)
	%	95% CI		%	95% CI		%	95% CI		
Sexual victimization without penetration perpetrated by										
Family member	11.07	10	12.13	12.86	11.72	14	11.93	10.83	13.03	2.54 (.111)
Teacher	8.13	7.18	9.07	7.98	7.05	8.91	8.06	7.12	8.99	.02 (.878)
Religious leader	8.08	7.13	9.03	7.2	6.3	8.1	7.66	6.74	8.59	.87 (.352)
Another known person	16.13	14.87	17.4	12.03	10.91	13.14	14.17	12.97	15.37	1.25 (.001)
Any known person	24.26	22.81	25.7	22.22	20.81	23.62	23.27	21.85	24.7	1.96 (.161)
Unknown person	8.51	7.55	9.48	8.77	7.79	9.75	8.64	7.66	9.61	.07 (.797)
Chi square** (*p*-value)	335.72 (<.0001)			519.05 (<.0001)			832.92 (<.0001)			
Happened to me before 18 years old	8.69	7.72	9.67	8.48	7.51	9.44	8.59	7.62	9.56	.048 (.827)
Any sexual victimization no penetration	27.21	25.71	28.7	24	22.57	25.44	25.66	24.19	27.13	4.55 (.033)
Sexual victimization with penetration perpetrated by										
Family member	6.97	6.08	7.86	5.1	4.33	5.87	6.08	5.25	6.92	4.77 (.029)
Teacher	5.05	4.29	5.82	4.64	3.9	5.38	4.86	4.1	5.61	.29 (.590)
Religious leader	5.18	4.4	5.97	5.15	4.37	5.93	5.17	4.39	5.95	.002 (.965)
Another known person	6.75	5.87	7.63	7.24	6.33	8.15	6.98	6.09	7.88	.29 (.588)
Any known person	13.11	11.94	14.28	11.76	10.64	12.88	12.46	11.32	13.61	1.32 (.25)
Unknown person	5.13	4.35	5.9	5.59	4.78	6.4	5.35	4.55	6.14	.32 (.57)
Chi square** (*p*-value)	564.20 (<.0001)			491.15 (<.0001)			1053.97 (<.0001)			
Happened to me before 18 years old	7.75	6.81	8.69	6.07	5.23	6.91	6.95	6.05	7.84	3.36 (.067)
Any sexual victimization with penetration	15.65	14.39	16.91	13.19	12.01	14.36	14.47	13.25	15.69	3.93 (.047)
Any sexual victimization perpetrated by										
Family member	14.06	12.88	15.23	13.42	12.26	14.57	13.75	12.58	14.91	.29 (.592)
Teacher	10.29	9.25	11.32	8.88	7.91	9.85	9.61	8.6	10.62	1.88 (.171)
Religious leader	10.5	9.45	11.56	9.08	8.09	10.07	9.82	8.8	10.85	1.84 (.174)
Another known person	17.98	16.67	19.29	14.56	13.35	15.76	16.34	15.07	17.6	7.05 (.008)
Any known person	25.75	24.28	27.23	24.22	22.78	25.66	25.01	23.56	26.47	1.06 (.302)
Unknown person	11.6	10.5	12.7	9.83	8.8	10.85	10.75	9.68	11.81	2.65 (.104)
Chi square** (*p*-value)	467.35 (<.0001)			505.20 (<.0001)			967.31 (<.0001)			
Happened to me before 18 years old	12.08	10.96	13.2	10.38	9.33	11.43	11.27	10.18	12.35	2.33 (.127)
Any sexual victimization	29.02	27.5	30.55	25.73	24.26	27.2	27.44	25.94	28.94	4.63 (.031)

*Note.* * Chi square determining difference between men and women for the prevalence of sexual victimization; **Chi square determining difference between sexual victimization perpetrated by Known person and by Unknown person.

Prevalence rates concerning child sexual violence were also analyzed. A total of 11.27% (95% CI 10.18–12.35) of participants reported they have experienced any form of sexual violence before 18: 8.59% (95% CI 7.62–9.56) of sexual violence without penetration and 6.95% (95% CI 6.05–7.84) with penetration. There is no significant difference among male and female participants for any form of child sexual violence (Table 2). Table 2 also presents results on perpetrators of sexual victimization. In any form considered, participants are significantly more likely to experience sexual violence from a known person than an unknown person.

Considering exposure to other overlap to other factors and forms of violence and victimization, about one out of two respondents in the overall sample reported experiences of family physical violence (50%), emotional abuse by mother (52%) and emotional abuse by father (50%) followed by physical violence (29%) and divorce of parents (18%). As presented in Table 3, all the aforementioned exposures to different forms of violence and violence were significantly associated with higher odds of reporting sexual victimization. When controlling for other factors (adjusted models), gender and emotional abuse by mother were no longer statistically significant; increased risk of sexual violence was noted among those who were exposed to the divorce of their parents (OR = 1.59, 95% CI 1.30–1.94; *p* < .0001), physical violence (OR = 2.09, 95% CI 1.75–2.50; *p* < .0001), family physical violence (OR = 1.51, 95% CI 1.27–1.79; *p* < .0001), and emotional abuse by father (OR = 1.35, 95% CI 1.12–1.63; *p* < .0001).

**Table 3.** Gender and other forms of violence as correlates of sexual violence.

		Prevalence of Sexual Victimization Across Other Indicators			Prevalence of Other Form of Victimization			Unadjusted OR			Adjusted^a^ OR			
		%	95% CI		%	95% CI		OR	95 CI		OR	95 CI		*p*-Value
Gender	Women	29.02	27.50	30.55	—			.85	.73	.99	.86	.72	1.01	.068
	Men	25.73	25.73	25.73										
Divorce of parents	Yes	37.28	35.64	38.91	18.37	17.11	19.63	1.77	1.47	2.13	1.59	1.30	1.94	<.0001
	No	25.08	23.62	26.55										
Physical victimization	Yes	41.07	39.40	42.74	28.58	27.10	30.06	2.48	2.11	2.92	2.09	1.75	2.50	<.0001
	No	21.95	20.54	23.35										
Family physical violence	Yes	33.19	31.59	34.79	50.40	48.75	52.05	1.79	1.54	2.10	1.51	1.27	1.79	<.0001
	No	21.69	20.29	23.09										
Emotional abuse by mother	Yes	28.47	26.92	30.01	51.62	50.01	53.23	1.16	1.00	1.36	.90	.74	1.09	.266
	No	25.48	23.99	26.98										
Emotional abuse by father	Yes	30.74	30.74	30.74	49.48	47.85	51.12	1.46	1.24	1.70	1.35	1.12	1.63	.002
	No	23.35	21.89	24.82										

^a^Controlling for gender, divorce of parents, physical victimization, family physical violence, emotional abuse by mother, emotional abuse by father.

Table 4 summarizes main risk factors associated to sexual violence using both unadjusted and adjusted models. Both models revealed significant associations with respondents’ gender, having children, education level, marital status, and living place (parents’ house, family member’s house, alone, etc.). Results from adjusted model showed that women (OR = .8; *p*=.002), older respondents (OR= 1.0; *p* = .03), those having children (OR = 1.5; *p* = .02), having graduate education (OR = 8.5; *p* = .02), being married (OR = 1.6; *p* = .01), and those living alone (OR = 3.7; *p* = .001) were more likely to report experiencing sexual victimization.

**Table 4.** ORs for risk and protective factors for any form of sexual victimization (*n* = 3944).

	Unadjusted OR				Adjusted^a^ OR			
	OR	95 CI		*p*-Value	OR	95 CI		*p*-Value
Gender	.85	.73	.99	.03	.78	.66	.91	.002
Age	1.03	1.00	1.06	.08	1.04	1.00	1.09	.028
Employment	1.14	.90	1.45	.29	1.00	.76	1.33	.980
Residence area	1.06	.91	1.23	.45	1.13	.91	1.40	.258
Having children	1.94	1.46	2.57	<.0001	1.52	1.09	2.14	.015
Education				.01				.029
Primary School	.39	.21	.73	<.0001	1.21	.53	2.76	.656
Incomplete Secondary School	.40	.21	.75	<.0001	1.12	.49	2.57	.796
Secondary School	.34	.18	.66	<.0001	.87	.37	2.04	.743
Incomplete university	.38	.20	.73	<.0001	.87	.37	2.07	.753
University	.36	.17	.75	.01	.79	.31	2.05	.632
Graduate	3.12	.56	17.26	.19	8.52	1.40	52.02	.020
Marital status				<.0001				<.0001
Married	1.76	1.27	2.45	<.0001	1.61	1.12	2.32	.010
Separated	1.13	.60	2.13	.71	1.02	.53	1.95	.957
Other	2.51	1.73	3.65	<.0001	2.54	1.71	3.79	.000
Living place				<.0001				.006
With a family member (uncle, aunty, etc.)	1.12	.91	1.38	.27				
Friend of the family	1.00	.68	1.48	1.00	.82	.53	1.25	.351
Child labor (domesticity)	1.30	.78	2.15	.32	1.33	.76	2.31	.318
Alone	4.17	2.03	8.56	.00	3.72	1.71	8.09	.001
In the street (Homeless)	12.85	2.70	61.08	.00	5.67	.91	35.46	.063
Orphanage	.82	.19	3.50	.78	.88	.20	3.82	.864
In a camp	10.25	1.98	53.16	.01	7.86	.87	70.61	.066

Reference categories are the following: Gender: Female; Employment: None; Residence area: Rural; Having children: No; Education level: None; Marital status: Single; Living place: Parents’ house.

^a^Controlling for gender, age, employment, residence area, having children, education level, marital status, living place.

Prevalence of mental health consequences (symptoms of PTSD, depression, psychological distress), and substance abuse are presented in Table 5. More than half of the participants who have reported sexual violence also reported substance abuse (53.8%, 95% CI 51.25–56.36), 54.92% (95% CI 52.4–57.44) for psychological distress symptoms, 62.63% (95% CI 60.15–65.12) for depression symptoms, and 40.1% (95% CI 37.67–42.52) for PTSD symptoms. Prevalence of substance abuse (41.52%, 95% CI 38.99–44.05), psychological distress (41.8%, 95% CI 39.33–44.33), depression (43.4%, 95% CI 40.85.-45.94), and PTSD (21.1%, 95% CI 19.08–23.12) were significantly lower among participants who have not reported sexual victimization. Results showed a strong association between sexual violence and mental health problems and substance abuse. Participants who reported having experienced sexual violence are more at risk to present to meet criteria of PTSD (OR = 1.96, 95% CI 1.66–2.32; *p* < .0001), depression (OR = 1.73, 95% CI 1.47–2.02; *p* < .0001), psychological distress (OR = 1.72, 95% CI 1.47–2.02; *p* < .0001), and use substance (OR = 1.33, 95% CI 1.13–1.57; *p* < .0001). Male participants who have experienced sexual violence are at greater risk to present mental health consequences and use substance relative to sexual violence experiences among girls and women, except for psychological distress. In addition to the above-mentioned results, separate models were estimated for each mental health problem and substance abuse considering sexual violence controlling for gender, age, employment, residence area, having children, education level, marital status, and living place. The adjusted odd ratios also confirmed the greater likelihood of reporting mental health problems and substance abuse among those who have experienced sexual violence.

**Table 5.** Mental health-related correlates of sexual victimization among young people by gender.

	No Lifetime Victimizationd			Lifetime Victimizationd			Total Population			Unadjusted OR					Adjusted^a^ OR			
	%	95% CI		%	95% CI		%	95% CI		OR	p-Value	95% CI			OR	p-Value	95% CI	
PTSD														1.96		<.0001	1.64	2.35
Men	21.1	19.08	23.12	40.1	37.67	42.52	25.86	24.41	27.3	2.51	<.0001	1.96	3.2					
Women	21.53	19.59	23.48	30.38	28.2	32.56	23.9	22.49	25.3	1.59	<.0001	1.26	2.01					
Total	21.32	19.92	22.72	34.74	33.11	36.37	24.84	23.41	26.26	1.96	<.0001	1.66	2.32					
Chi square* (*p*-value)	.07 (.795)			9.28 (.002)			1.82 (.178)											
Depression														1.6		<.0001	1.35	1.9
Men	39.06	36.55	41.56	56.84	54.29	59.38	42.98	40.51	45.45	2.06	<.0001	1.62	2.61					
Women	38.64	36.27	41.02	45.13	42.71	47.56	39.98	37.67	42.28	1.31	.015	1.05	1.62					
Total	38.85	37.12	40.57	50.29	48.52	52.06	41.39	39.7	43.09	1.59	<.0001	1.36	1.87					
Chi square* (*p*-value)	.04 (.842)			11.53 (.001)			3.03 (.082)											
Psychological distress														1.52		<.0001	1.33	1.87
Men	41.83	39.33	44.33	54.92	52.4	57.44	44.44	42.76	46.12	1.7	<.0001	1.35	2.14					
Women	35.5	33.17	37.84	49.57	47.13	52.01	38.89	37.24	40.53	1.78	<.0001	1.43	2.22					
Total	38.61	36.9	40.32	52	50.25	53.76	41.57	39.9	43.23	1.72	<.0001	1.47	2.02					
Chi square* (*p*-value)	9.57 (.002)			2.42 (.120)			10.68 (.001)											
Substance abuse														1.22		.034	1.02	1.46
Men	41.52	38.99	44.05	53.8	51.25	56.36	44.44	42.74	46.14	1.64	<.0001	1.3	2.09					
Women	64	61.63	66.36	64.97	62.62	67.31	63.65	62	65.3	1.04	.714	.83	1.31					
Total	52.99	51.21	54.76	59.95	58.21	61.69	54.41	52.71	56.12	1.33	.001	1.13	1.57					
Chi square* (*p*-value)	112.85 (<.0001)			10.52 (.001)			121.54 (<.0001)											

*Note.* * Chi square assessing difference between men and women.

^a^Controlling for gender, age, employment, residence area, having children, education level, marital status, living place, divorce of parents, physical victimization, family physical violence, emotional abuse by mother, emotional abuse by father.

## Discussion

This is the first study relying on a representative sample of adolescents and men and women young adults that examined the prevalence, risk factors, and mental health consequences associated with sexual victimization, both in Haiti and throughout the Caribbean. Prior to this study, detailed data was not available on sexual violence among youth before and after the age of 18, nor on its associated risk factors and mental health consequences. Following studies conducted in North America (Finkelhor et al., 2014; Hébert et al., 2019; Pérez-Fuentes et al., 2013) and Europe (Hébert et al., 2020; Jonas et al., 2011; Sanjeevi et al., 2018), more studies with nationally representative samples have been conducted on these issues in Africa (Breiding et al., 2011; Gladden et al., 2011; Reza et al., 2009; United Nations Children’s, 2012; Ward et al., 2018) and Asia (Choudhry et al., 2018; Ma, 2018). Apart from the DHSs that includes a non-representative sample of adolescents and young adults and a few studies conducted among children, significant gaps persisted in the Caribbean and more particularly in Haiti (Debowska et al., 2018; Decker et al., 2015; Descartes et al., 2020; Lai et al., 2020).

The findings showed that sexual violence is a public health emergency in Haiti. More than one out of four adolescents and young adults have experienced at least one form of sexual violence (27.44%). Adolescent girls and young women (29.02%) were more likely than adolescent boys and young men (25.73%) to experience sexual victimization. Specifically, 25.66% of participants experienced sexual violence without penetration, that is, involving touching (27.21% of female adolescents and 24% of male adolescents), while 14.47% experienced sexual violence involving penetration (15.65% of female adolescents and 13.19% of male adolescents). Data collected in the Violence Against Children Surveys following the 2010 earthquake in Haiti showed lower prevalence of sexual violence in both female adolescents and young women (23.01%) and male adolescents and young men (15.98%) aged 13 to 23 (Lai et al., 2020).

The prevalence of child sexual violence is 11.27% (12.09% for girls and 10.38% for boys). The total prevalence is slightly lower than the global child sexual violence estimates of 12.7% (Stoltenborgh et al., 2011). Compared to our data, this same study indicates that prevalence was higher among female samples (18.0%) and lower among male samples (7.6%) (Stoltenborgh et al., 2011). However, when considering the combined prevalence in low- and middle-income countries similar to Haiti among adolescents and young adults, the prevalence of child sexual violence is higher for both female and male samples (15.9% and 14.0%, respectively). The same observation can be made in other representative sample studies in other low- and middle-income countries such as Tanzania, Kenya, and Swaziland (Breiding et al., 2011; Gladden et al., 2011; Reza et al., 2009; United Nations Children’s, 2012). It is also important to note that there are no significant differences between girls and boys for child sexual violence as opposed to lifetime sexual victimization. While this may seem surprising given the vulnerability often observed among girls in Haiti, a publication using the Violence Against Children Surveys data among 18–24 year olds found nearly similar prevalence of child sexual violence with penetration between girls (9%) and boys (7.6%) (Sumner et al., 2015). These findings also offer a better perspective on adolescence and emerging adulthood, which appear to be particularly high-risk periods during which gender inequalities seem to be accentuated.

This study confirms the observation that has been made in both high-income and low- and middle-income countries, that more often than not, victims are victimized by someone they know, including individuals in a position of authority or caregiver role (family members, teachers, religious leaders) (Mwangi et al., 2015; Reza et al., 2009; Yahaya et al., 2012). This study also reveals a set of risk factors for victims of sexual violence in Haiti including parental divorce, physical victimization, physical violence within the family, and emotional abuse perpetrated by father and mother. Studies conducted in different contexts have shown similar risk factors for sexual violence and have highlighted the high co-occurrence of different forms of violence (Finkelhor, 1994; Ward et al., 2018). These risk factors can also be analyzed by taking into consideration the fact that perpetrators are often aware of the vulnerabilities faced by these youth. It is often family members, teachers, religious leaders, and people with significant social influence who abused these youth. Furthermore, in Haiti, these results provide important insights into important social aspects, as physical punishment, bullying, verbal victimization, and degrading language toward children continue to be the preferred means of “educating” into the families (Cénat, Derivois, et al., 2018; Flynn-O’Brien et al., 2016; Institut HaÏtien de l’Enfance -IHE, 2018). Other important sociodemographic risk factors were also revealed including having children, being married, having a university education, living alone, living in camps, and living on the street. A mixed-methods study conducted in Haiti revealed how street children are particularly at risk of experiencing various forms of sexual victimization, often by Westerners who go to Haiti for the purpose of having pedophilia sex (Cénat, Derivois, et al., 2018). For young people who have children and are married, often those children and the marriage are the result of sexual violence (Kopelman, 2016; Ouattara et al., 1998). Instead of legal action being taken, the adolescent girls and young women were offered as wives to the perpetrators of the rape, in a total violation of their rights. Having a university level of education was found to be an important risk of being a victim of sexual violence. This observation corroborates results from a study conducted in Haiti that has already shown that women with a university level of education were among the most likely to experience sexual violence (Gage & Hutchinson, 2006). However, most studies do not address this issue and when it is addressed, only a basic level of education is analyzed. More specific studies need to be conducted to determine the perpetrators and what risk factors are associated.

Lastly, this study revealed a strong association between sexual violence and mental health problems and substance abuse. The results showed that victims of sexual violence are 2.32 times more likely to meet criteria of PTSD, 2.02 times more likely to have depression and psychological distress, and 1.57 times more likely to use substance. The results also showed that these risks are higher for adolescent boys and young men who have experienced sexual violence as opposed to adolescent girls and young women. These differences may be explained by the taboo nature of sexual violence experienced by men in Haiti. Although this study shows that this is not an isolated issue, but rather a major public health problem in which more than one in four adolescents or young men in Haiti are victims (25.73%), it remains a taboo subject rarely discussed in society (Cénat et al., 2020). Although the strong association between sexual violence and mental health problems is already known, these findings are critically important in Haiti, as the country has very little mental health infrastructure, all of which are concentrated in the Capital region Port-au-Prince, and with very few mental health professionals (less than 40 psychiatrists, less than 200 psychologists, less than 400 social workers for the entire country) (Cénat et al., 2021). In addition, mental health issues are still stigmatized and less than 10% of victims of child sexual violence received health services following their violence (Sumner et al., 2015). These considerations demonstrate that the prevalence of sexual violence and its consequences constitute a public health emergency.

This study has a few limitations. Firstly, although it is a representative sample, the cross-sectional nature of the data prevents making causal links between sexual violence and mental health problems. A longitudinal design in victim care services is needed to examine links to mental and physical health problems. Secondly, lifetime sexual violence and child sexual violence were assessed retrospectively, soliciting participants’ memories that may be defective and minimizing instances of sexual victimization, especially during childhood. Thirdly, given the literacy rate and for logistical issues of study validation, the interviewers completed the questionnaires themselves by questioning the participants. Although the interviewers were appropriately trained in cultural, gender and educational issues, this may also have inadvertently discouraged some participants from reporting their victimization. Finally, we did not integrate sexual abuse perpetrated by partners. This is an important limitation given that many adolescent girls and young women experience sexual violence from their partners.

Despite these limitations, this study, which is the first of its kind in the Caribbean, provides important information on the prevalence and consequences associated with sexual violence among adolescents and young adults in Haiti. It shows that sexual violence is a major public health emergency. Similar research should be conducted in other Caribbean countries to develop a regional trend that can facilitate the mutualization of resources for the development of prevention and intervention programs. In the meantime, this study provides the necessary data for the development of a national policy for sexual violence prevention and intervention. It also provides results to facilitate, motivate, and argue for the implementation of new laws to facilitate care-seeking, complaint filing, victim protection, and perpetrator legislation.
